# Venous Thrombosis and Thrombocyte Activity in Zebrafish Models of Quantitative and Qualitative Fibrinogen Disorders

**DOI:** 10.3390/ijms22020655

**Published:** 2021-01-11

**Authors:** Richard J. Fish, Cristina Freire, Corinne Di Sanza, Marguerite Neerman-Arbez

**Affiliations:** Department of Genetic Medicine and Development, Faculty of Medicine, University of Geneva, 1211 Geneva, Switzerland; Richard.Fish@unige.ch (R.J.F.); cristinafreiresanz@gmail.com (C.F.); corinne.disanza@unige.ch (C.D.S.)

**Keywords:** fibrinogen, fibrin, thrombocytes, thrombosis, zebrafish

## Abstract

Venous thrombosis occurs in patients with quantitative and qualitative fibrinogen disorders. Injury-induced thrombosis in zebrafish larvae has been used to model human coagulopathies. We aimed to determine whether zebrafish models of afibrinogenemia and dysfibrinogenemia have different thrombotic phenotypes. Laser injuries were used to induce venous thrombosis and the time-to-occlusion (TTO) and the binding and aggregation of fluorescent *Tg(itga2b:EGFP)* thrombocytes measured. The *fga^−/−^* larvae failed to support occlusive venous thrombosis and showed reduced thrombocyte binding and aggregation at injury sites. The *fga^+/−^* larvae were largely unaffected. When genome editing zebrafish to produce fibrinogen Aα R28C, equivalent to the human Aα R35C dysfibrinogenemia mutation, we detected in-frame skipping of exon 2 in the fga mRNA, thereby encoding Aα^Δ*19–56*^. This mutation is similar to Fibrinogen Montpellier II which causes hypodysfibrinogenemia. Aα^*+/*Δ*19–56*^ fish had prolonged TTO and reduced thrombocyte activity, a dominant effect of the mutation. Finally, we used transgenic expression of fga R28C cDNA in fga knock-down or *fga^−/−^* mutants to model thrombosis in dysfibrinogenemia. Aα R28C expression had similar effects on TTO and thrombocyte activity as Aα^*+/*Δ*19–56*^. We conclude that thrombosis assays in larval zebrafish can distinguish between quantitative and qualitative fibrinogen disorder models and may assist in anticipating a thrombotic phenotype of novel fibrinogen mutations.

## 1. Introduction

Mutations in the three fibrinogen genes give rise to congenital fibrinogen disorders [1]. Fibrinogen has a crucial role as the soluble fibrin precursor in blood clotting, and its deficiency or dysfunction manifests in both bleeding and thrombotic clinical events in these disorders [2,3]. Two copies of three protein chains make up the soluble fibrinogen hexamer, (AαBβγ)_2_, with each chain encoded by its respective gene (*FGA*, *FGB* or *FGG*) that is expressed in the liver. Mutations in any of the genes can lead to a quantitative or qualitative fibrinogen disorder. Quantitative disorders include afibrinogenemia, where plasma fibrinogen antigen is undetectable, and hypofibrinogenemia where fibrinogen is below the typical healthy range (1.5–4 g/L). The qualitative disorders are dysfibrinogenemia, where a normal antigen level is accompanied by a lower functional level of fibrinogen when compared to control plasma in clotting assays, and hypodysfibrinogenemia where an abnormally low antigen level is discordant with an even lower functional activity [4].

Afibrinogenemia shows recessive inheritance, two mutated alleles of a fibrinogen gene are required for its appearance, and hypofibrinogenemia can be detected in heterozygous carriers of alleles that would cause afibrinogenemia in homozygosity [5]. Dysfibrinogenemia and hypodysfibrinogenemia show dominant inheritance and are typically caused by heterozygous missense mutations [6]. Quantitative and qualitative fibrinogen disorders are rare [7] and patients can suffer from both bleeding and thrombosis. Put together, this creates a particular therapeutic difficulty where maintenance of hemostasis and prevention of thrombosis may need to be balanced in a patient group for which reports of optimal treatments are scarce [6,8].

While diagnosis can usually be achieved by laboratory tests and genetic studies of the fibrinogen genes, prediction of the clinical phenotype of fibrinogen disorders beyond a bleeding tendency is challenging [2]. Familial histories can be informative, but different family members with the same causative mutation may display bleeding or thrombosis (for example [9]). Therefore, a complement of additional tests, including global hemostasis assays and fibrin clot analysis, can offer a more complete predictive picture of the propensity of a given mutation to lead to an adverse clinical outcome [10,11].

Bleeding events linked to low plasma fibrinogen, or a dysfunctional fibrinogen molecule, can be explained by a deficiency in the quantity or quality of the major physiological substrate for coagulation-based clotting. While thrombosis in afibrinogenemia and dysfibrinogenemia may seem paradoxical, several mechanisms have been proposed. The binding of thrombin to fibrin is the basis of fibrin’s antithrombin I activity [12,13]. Fibrin sequesters thrombin, but if fibrin is absent [14], or thrombin binding is affected [15], this potentially releases thrombin activity. This may increase activation of platelets and their aggregation mediated by von Willebrand Factor [16]. Fibrinogen can also inhibit platelet binding to surface-bound fibrinogen [17], suggesting that platelet adhesion to a vessel wall could be enhanced by a reduction of soluble fibrinogen. Dysfibrinogenemia mutations which cause altered clot structure and affect plasminogen binding can lead to impaired fibrinolysis [18,19], a further possible contributor to thrombosis. This panel of possibilities helps explain the prevalence of thrombosis in fibrinogen disorders but also implies that it is fibrin(ogen) quantity and quality that influence it.

In order to study the pathophysiology of coagulation disorders, animal models have been extensively employed, especially mice [20]. This spans from rare bleeding disorders through a spectrum of thrombosis models [21]. The zebrafish, *Danio rerio*, has emerged as an alternative animal model in thrombosis and hemostasis research. Initially it was proposed due to its conserved vertebrate fibrin-based clotting mechanism [22] and amenability to genetic screening that could unveil novel factors in hemostasis [23]. The model’s major strength is a combination of high fecundity, accessibility of near transparent larval tissues and blood vessels that can be observed and manipulated in real time, and robust tools to make genetic modifications [24]. This has been demonstrated in a number of recent models of hemostasis and coagulation factor deficiencies that recapitulate phenotypes found in patients and enable screening of potential pathogenic variants [25,26,27,28,29,30,31].

Rather than platelets, like all teleosts, zebrafish have thrombocytes [32]. These arise about two days into embryonic zebrafish development but are only circulating in sufficient numbers to contribute to hemostasis and thrombosis after about four days. Thrombocytes bear many features of mammalian platelets, and are considered their functional equivalents despite being considerably larger and retaining a nucleus [33]. The developmental time period when transparent larval zebrafish are particularly useful for observing and challenging the hemostatic system, at 3 to 5 days postfertilization, spans a shift from a circulatory system that is almost devoid of thrombocytes, to one where thrombocytes are present and can play an active role in hemostasis and thrombosis.

In the present study, our goal was to assess the experimental thrombotic response to a venous laser injury [34] in larval zebrafish models of afibrinogenemia and dysfibrinogenemia. We aimed to understand whether assays in zebrafish larvae could distinguish between models phenotypically, as a possible future functional in vivo read-out for novel mutations identified in patients with fibrinogen disorders.

Afibrinogenemic larvae (*fga^−/−^*) failed to support venous thrombosis, as described previously [27,35], and had markedly reduced thrombocyte binding and accumulation at sites of injury, with aggregates showing a tendency to embolize. In preparing a zebrafish line designed to model a dysfibrinogenemia-causing missense mutation, we detected fga transcripts with exon 2 skipping and an mRNA encoding an Aα chain resembling a fibrinogen found in hypodysfibrinogenemia [9]. We used morpholino knock-down of fga mRNA or *fga* mutants, with transgenic expression of Aα R28C cDNA, to model a human dysfibrinogenemia. Both the dysfibrinogenemia and hypodysfibrinogenemia models gave prolonged venous thrombosis times and reduced thrombocyte activity as dominant effects seen in heterozygotes. Laser-induced thrombosis in zebrafish can therefore distinguish between models of quantitative and qualitative fibrinogen disorders, particularly with respect to thrombocyte activity, the inheritance mode of the disorder, and provide a direct demonstration of the thromboembolic nature of fibrinogen deficiencies.

## 2. Results

### 2.1. Afibrinogenemic Zebrafish Larvae Fail to Support Venous Thrombosis and Have Impaired Thrombocyte Adhesion and Aggregation at Injury Sites

**Figure 1:**
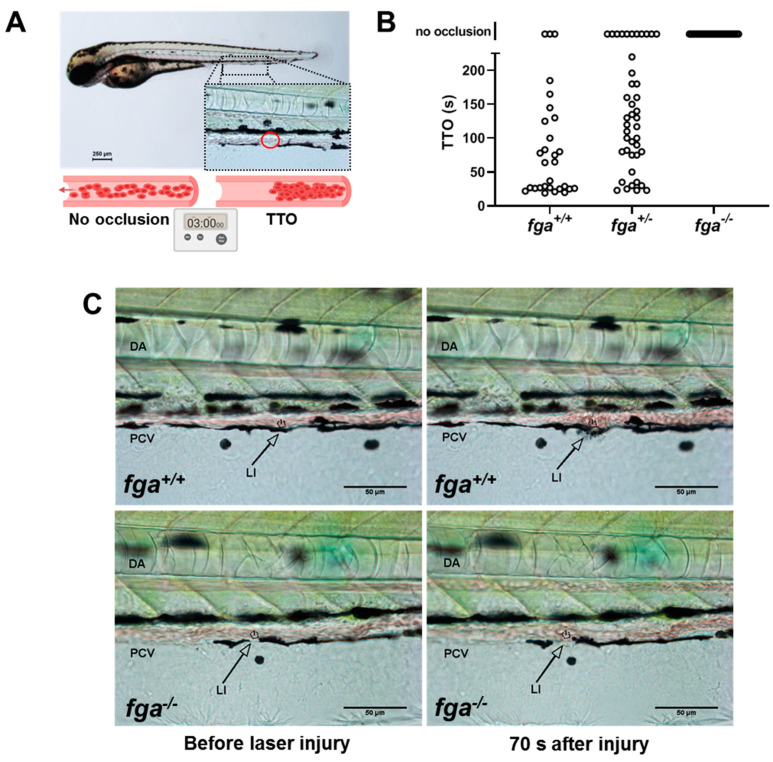
Laser-induced venous thrombosis in an afibrinogenemia model. (**A**) Diagram of time-to-occlusion (TTO) assay. The posterior caudal vein (PCV) is targeted for laser injury (region represented by the red circle) near the 5th somite distal to the cloaca and the time to PCV occlusion measured. A 3-min cut-off is used to determine no occlusion. (**B**) TTO in seconds (s) in 3 dpf *fga^+/+^*, *fga^+/−^* and *fga^−/−^* larvae. Each circle represents an individual larva. (**C**) Time lapse images of 3 dpf *fga^+/+^* and *fga^−/−^* larvae before and after laser injury at the position marked (LI). DA is the dorsal aorta.

Laser injury of the posterior cardinal vein in 3-day postfertilization (3 dpf) zebrafish larvae can lead to occlusive venous thrombosis and when monitored gives the time-to-occlusion (TTO, Figure 1A). We used this technique in fibrinogen mutants to test whether the absence of fibrinogen alters the TTO. As we reported previously [35] and in similar mutants [27], injury in *fga^−/−^* larvae did not lead to occlusion (Figure 1B,C). Of the larvae supporting occlusion the mean TTO was 72 s in *fga^+/+^* and 114 s in *fga^+/−^* animals, respectively (Figure 1B). We include this replication of previous work in the current study to provide images (Figure 1C) and short videos not reported previously. Examples of laser injuries in *fga^+/+^* and *fga^−/−^* are shown in in Supplementary Videos S1 and S2, respectively. For scaling of these Supplementary Videos, compare to Figure 1C.

**Figure 2:**
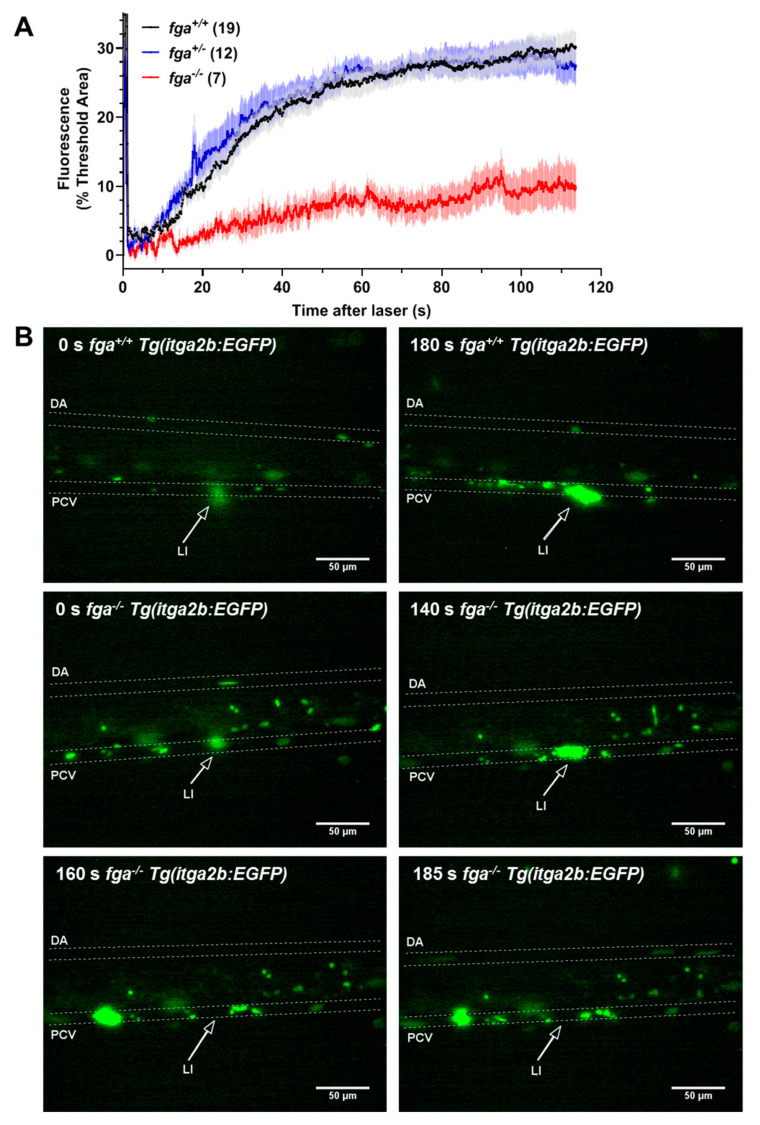
Thrombocyte adhesion and aggregation in afibrinogenemic zebrafish. (**A**) Laser-induced vessel injury to measure thrombocyte binding and accumulation in 5 dpf *Tg(itga2b:EGFP)* embryos with fluorescent thrombocytes. The thrombocyte-associated green fluorescence accumulation after laser injury within a defined region was measured over time for individual larvae. Each line represents the mean fluorescence (+/− SEM) for each group. The number of larvae per group is indicated in brackets. (**B**) Images from *fga^+/+^* and *fga^−/−^* larvae upon (0 s) and after laser injury, times as indicated. The dorsal aorta (DA), posterior cardinal vein (PCV) and site of laser injury (LI, arrow) are indicated. Green near the laser injury site (arrow head) indicates the laser flash (0 s) and the accumulation of green fluorescent thrombocytes at later time points.

The adhesion and aggregation of fluorescent thrombocytes was assessed after laser injury of the PCV in 5 dpf *Tg(itga2b:EGFP) fga^+/+^*, *fga^+/−^*, and *fga^−/−^* larvae. Thrombocyte binding and accumulation was hindered in *fga^−/−^* larvae compared to *fga^+/+^* or *fga^+/−^*. Fluorescence curves for *fga^+/+^* and *fga^+/−^* superimposed (Figure 2A). Two minutes after laser injury, *fga^−/−^* larvae typically had few bound thrombocytes at the injury site and showed a clear tendency for embolism of clumps of fluorescent cells after initial adhesion at the injury site (Figure 2B). Thrombocyte aggregation data were reported previously for *Tg(itga2b:EGFP) fga^+/+^* and *fga^−/−^* genotypes [35], but not compared to *fga^+/−^*. We made this replication in order to present the *fga^+/−^* larvae, the embolic phenotype seen in *fga^−/−^*, and to provide images (Figure 2B) and videos of the result that were not used previously. Thrombocyte activity after PCV laser injuries is illustrated in Supplementary Video S3 (*fga^+/+^*) and 4 (*fga^−/−^*). For scaling of Supplementary Videos, compare to Figure 2B.

### 2.2. Genome Editing of fga Exon 2

A frequently detected missense mutation found in dysfibrinogenemia patients is the *FGA* c.103C→T variant [36], leading to the fibrinogen Aα chain R35C amino acid change. Aα R35 (R16 in the mature protein, after signal peptide cleavage) is part of the Aα chain thrombin cleavage site. When present with the nonmutated Aα chain, Aα R35C leads to prolonged fibrin polymerization, delayed fibrinopeptide-A release, decreased binding to platelets, fibrinolytic resistance, and disordered fibrin networks [37].

**Figure 3:**
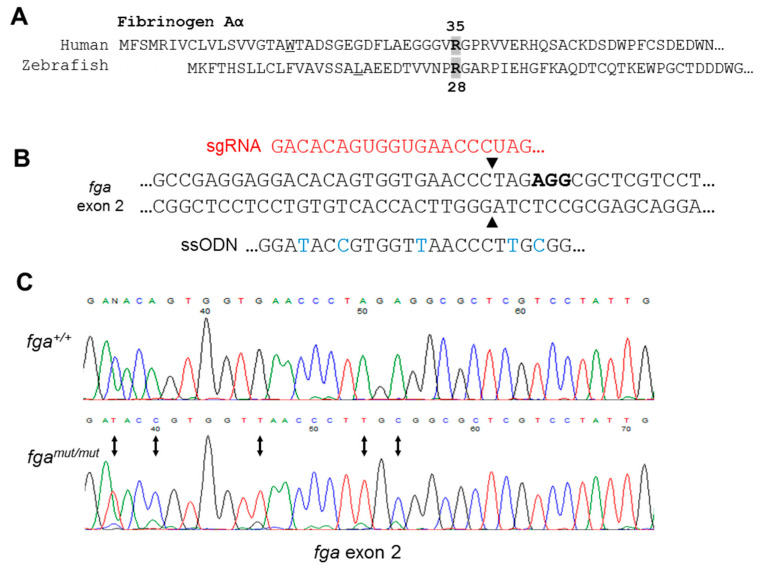
Genome editing of *fga* exon 2. (**A**) Aligned N-terminal region of human and zebrafish fibrinogen Aα chains. Aα R35 and Aα R28 are indicated. (**B**) CRISPR/Cas9 strategy for *fga* exon 2 editing through homology directed repair (HDR). In red is part of the sgRNA targeting the edited region by complementarity, in bold is the PAM sequence. Part of the ssODN for HDR is shown to highlight edited nucleotides (blue), including codon usage changes and the Arg to Cys codon change. (**C**) Sanger sequencing to confirm genome edited sequence in genomic DNA from homozygous mutant fish (*fga^mut/mut^*), compared to wild-type (*fga^+/+^*). Changed nucleotides are highlighted with arrows.

We aimed to produce zebrafish with a mutation equivalent to human Aα R35C. This corresponds to R28C in the zebrafish Aα (Figure 3A). Thrombin-catalyzed fibrinopeptide A (FpA) release does not occur in the human Aα R35C chain. To verify that zebrafish Aα R28C also prevents thrombin cleavage, we transfected HEK-293T cells with plasmids for expression of wild-type or Aα R28C zebrafish fibrinogen. Cell lysates and conditioned medium were treated with and without human thrombin and FpA and fibrinopeptide B (FpB) cleavage verified by immunoblotting with anti-zebrafish fibrinogen Aα and Bβ chain antibodies, respectively. Size shifts were seen in the zebrafish Aα and Bβ chain with thrombin treatment but not in the Aα R28C-containing fibrinogen, confirming its resistance to thrombin cleavage and FpA release (Supplementary Figure S1).

On the TU zebrafish genetic background, we used CRISPR-Cas9-based genome editing to induce double-stranded genomic DNA breaks near the R28 codon in exon 2 of the zebrafish *fga* gene. By microinjection of early zebrafish embryos with recombinant Cas9 nuclease, a single guide RNA targeting the region, and an oligonucleotide (ssODN) with the desired codon change as a template for homology-directed repair (HDR), in subsequently raised fish we identified a founder animal that transmitted the expected mutated *fga* allele to its offspring. The HDR oligonucleotide also contained 3 nucleotide changes to avoid retargeting of the mutated allele with the CRISPR-Cas9 reagents (Figure 3B). These changes conserved the Aα chain amino acid sequence and introduced an endonuclease restriction site to facilitate PCR-based genotyping (Supplementary Figure S2A). Confirmation of the mutated (*mut*) allele DNA sequence is shown in Figure 3C.

### 2.3. Exon 2 Skipping in fga mRNA after Genome Editing

**Figure 4:**
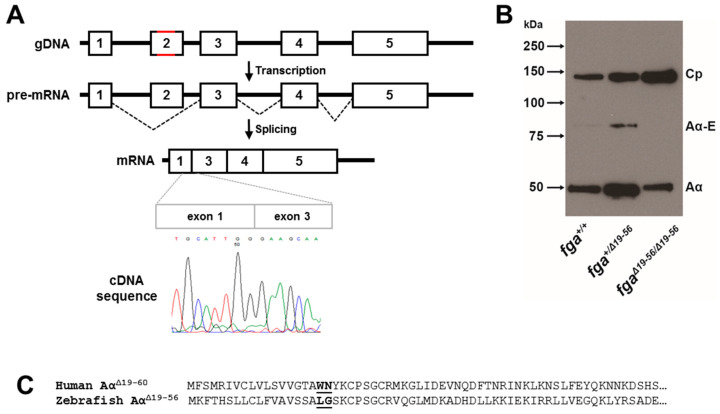
fga exon 2 skipping. (**A**) A scheme to highlight changes in *fga* exon 2 genomic DNA in red (gDNA) led to exon 2 skipping after transcription and splicing. A partial cDNA sequence to show the exon 1 to exon 3 splicing is shown. (**B**) Immunoblotting of plasma samples from *fga^+/+^*, *fga*^*+/Δ19–56*^, and *fga*^Δ*19–56/*Δ*19–56*^ fish with anti-ceruloplasmin and anti-fibrinogen Aα chain antibodies. (**C**) Amino acid alignment of the predicted fibrinogen Aα chains resulting from the human *FGA* IVS2+3insCAT mutation and the zebrafish fga^Δ*19–56*^ mRNA. The junction of exon 1- and exon 3-encoded amino acids are in bold and underlined for each chain.

To confirm the expected mutation at the RNA level, we generated cDNA from liver RNA extracted from adult *fga^+/+^*, *fga^+/mut^*, and *fga^mut/mut^* fish. Shorter than expected fga amplicon RT-PCR products were detected in cDNA from *fga^+/mut^*, and *fga^mut/mut^* fish, corresponding to shortened Aα and AαE transcripts (Supplementary Figure S2). Analysis of the fga cDNA sequences, by Sanger sequencing, revealed exon 2 skipping in mRNA from the *fga^mut^* allele (Figure 4A). We did not detect exon 2 sequences in RNA transcribed from the mutated allele when RT-PCR products were cloned and multiple clones analyzed (49 clones in total, data not shown). Instead of Aα R28C, the predicted Aα chain translated from this exon 2-skipped transcript lacks amino acids 19 to 56 but retains the natural open reading frame, encoding Aα Δ19–56.

To understand why the genome edited sequence led to exon 2 skipping in fga mRNA, we tested the hypothesis that it was affecting an exon splicing enhancer sequence (ESES), using prediction software (RESCUE-ESE Web Server—genes.mit.edu). The software predicted disruption of several ESESs in the mutated sequence (Supplementary Figure S3) providing a possible source for changes in fga mRNA splicing, including exon skipping [38].

We named the *fga^mut^* allele *fga*^Δ*19–56*^, for clarity. To assess whether mutant fibrinogen containing the Aα Δ19–56 chain was present in the zebrafish circulation, plasma from adult *fga^+/+^*, *fga*^+/Δ*19–56*^, and *fga*^Δ*19–56/*Δ*19–56*^ fish was subjected to immunoblotting, with ceruloplasmin as a plasma protein control. Fibrinogen Aα was present in *fga*^Δ*19–56/*Δ*19–56*^ plasma but, when normalized with ceruloplasmin, maybe at lower levels than in *fga^+/+^* and *fga*^*+/*Δ*19–56*^ (Figure 4B). This lower level may explain our inability to detect the AαE chain in *fga*^Δ*19–56/*Δ*19–56*^ plasma. In contrast to the situation in larvae [35], the Aα chain is detected more abundantly than the AαE chain in adult zebrafish plasma (RJF, MNA, unpublished). As this immunoblotting was intended to determine the presence or absence of mutated fibrinogen, sampling was not repeated, and relative band quantifications are not reported with *n* = 1.

We did not detect a size shift in the Aα Δ19–56 immunoblot compared to wild-type animals, despite a predicted loss of approximately 4.3kDa and a gel resolution that we would have expected to resolve this difference. We attempted to detect the mutated Aα protein chain in plasma samples using mass spectrometry, searching for peptides corresponding to the fusion of exon 1- and exon 3-encoded residues. We did not detect such peptide species, but an exon 2-encoded peptide (EWPGCTDDDWGSK) was detected in wild-type *fga^+/+^* and not *fga*^Δ*19–56/*Δ*19–56*^ plasma (Supplementary Figure S4). We conclude that the mutated Aα chain is present in plasma but currently have no explanation for the small discrepancy in predicted relative molecular mass seen by immunoblotting.

The change in the zebrafish Aα Δ19–56 protein sequence, compared to wild-type, resembles closely the Aα chain which is expressed as a result of a human splice-site mutation detected in a family with hypodysfibrinogenemia [9]. Fibrinogen Montpellier II, caused by the *FGA* IVS2 + 3insCAT mutation, lacks human Aα chain residues encoded by *FGA* exon 2. This Aα-chain is also devoid of a thrombin cleavage site and leads to detrimental effects on clot structure with thinner fibers and increased fiber ends, presumably due to the abnormal unpolymerized chain ends formed in the protofibril. An alignment of the amino-termini of the Aα chain in Fibrinogen Montpellier II (Aα Δ19−60) and zebrafish Aα Δ19–56 is shown in Figure 4C. With this similarity, we decided to characterize the venous thrombosis phenotype in zebrafish expressing Aα Δ19–56, as a model of hypodysfibrinogenemia.

### 2.4. Venous Thrombosis in Aα *Δ*19–56-Expressing Zebrafish Larvae

**Figure 5:**
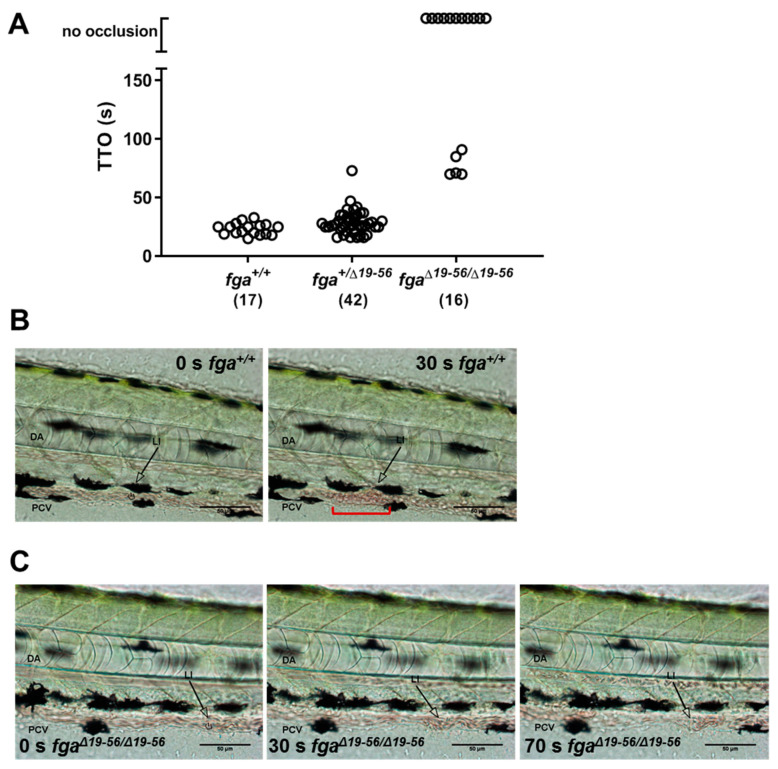
Laser-induced venous thrombosis in a hypodysfibrinogenemia model. (**A**) TTO for 3 dpf *fga^+/+^*, *fga*^*+/*Δ*19–56*^ and *fga*^Δ*19–56/*Δ*19–56*^ larvae. Each circle represents one larva. The number of larvae tested in each group is shown in brackets. (**B**,**C**) Time lapse images of *fga^+/+^* and *fga*^Δ*19–56/*Δ*19–56*^ larvae upon (0 s) or 30 s after injury. The dorsal aorta (DA), posterior cardinal vein (PCV) and site of laser injury (LI) are indicated. An arrow marks the laser target. A red bracket in the right panel of B highlights the region of thrombus formation.

We compared laser-induced TTO in the PCV of 3 dpf *fga^+/+^*, *fga*^*+/*Δ*19–56*^ and *fga*^Δ*19–56/*Δ*19–56*^ larvae. In contrast to *fga^+/+^* larvae from the AB background (Figure 1B) used for generating *fga*^−/−^, we recorded a relatively short TTO for all *fga^+/+^* fish with the TU background (mean 23 s, Figure 5A). This difference has been investigated in other work and relates to the ability to express the predominant larval fibrinogen alpha chain isoform (AαE) in larvae [35]. All *fga*^*+/*Δ*19–56*^ larvae also supported vessel occlusion after injury with a mean TTO of 29 s. A two-tailed unpaired *t*-test between TTOs measured for *fga^+/+^* (*n* = 17) versus *fga*^+/Δ19–56^ (*n* = 42) gave a *p*-value of 0.361. No occlusion was measured in 11 of 16 *fga*^Δ*19–56/*Δ*19–56*^ larvae. The mean TTO in the remaining 5 *fga*^Δ*19–56/*Δ*19–56*^ larvae that supported occlusion was 77 s. The *t*-tests comparing the TTO from this sub-group of 5 *fga*^Δ*19–56/*Δ*19–56*^ larvae and the *fga^+/+^* or *fga*^*+/*Δ*19–56*^ larvae gave *p*-values of <0.0001 for both analyses. We conclude that the presence of a single *fga*^*+/*Δ*19–56*^ allele has little effect on laser-induced TTO, compared to wild-type, but homozygous *fga*^Δ*19–56/*Δ*19–56*^ larvae either fail to show vessel occlusion or display a significant prolongation. Figure 5B shows vessel occlusion recorded 30 s postinjury in *fga^+/+^* larvae. A typical *fga*^Δ*19–56/*Δ*19–56*^ result is shown in Figure 5C where occlusion was not reached after 70 s.

In addition to differences in TTO, we observed qualitative differences in the clots formed in *fga*^Δ*19–56/*Δ*19–56*^ larvae, compared to *fga^+/+^* controls. When occlusion occurred in *fga*^Δ*19–56/*Δ*19–56*^ larvae it was short-lived and accompanied by visible clumping of erythrocytes in the vasculature. This difference can be seen by comparing Supplementary Video S5 (*fga^+/+^*) and Supplementary Video S6 (*fga*^Δ*19–56/*Δ*19–56*^). Even in *fga*^Δ*19–56/*Δ*19–56*^ larvae, where a TTO could not be recorded (no occlusion), cellular clumping arose and then resolved in the minutes after injury (Supplementary Video S7). For video scaling, compare to Figure 5B,C.

**Figure 6:**
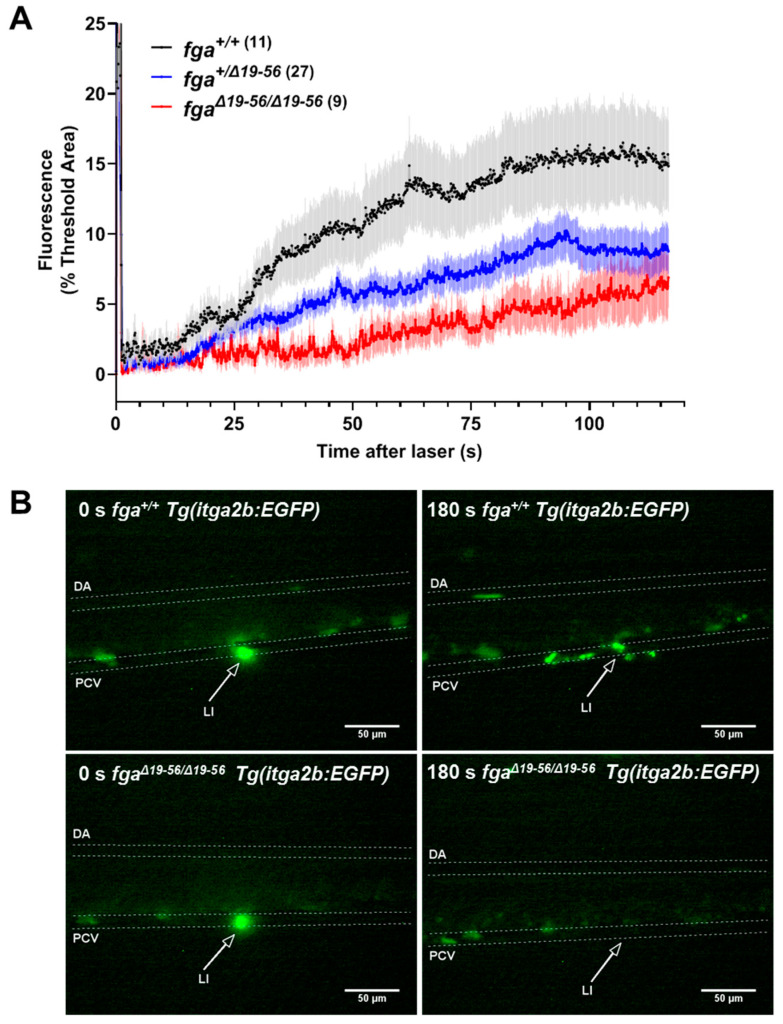
Thrombocyte adhesion and aggregation in a hypodysfibrinogenemia model. (**A**) Fluorescent thrombocyte binding and adhesion in 5 dpf *Tg(itga2b:EGFP)* embryos after laser injury of the PCV over time in *fga*^+/+^, *fga*^*+/*Δ*19–56*^ and *fga*^Δ*19–56/*Δ*19–56*^ genotypes. Each line represents the mean (+/−SEM) of thrombocyte fluorescence reaching a threshold fluorescence within a defined area. (**B**) Time lapse images of *fga*^+/+^ and *fga*^Δ*19–56/*Δ*19–56*^ larvae upon (0 s) and 180 s after laser injury. Fluorescence from the laser is seen at 0 s and where green fluorescent thrombocytes accumulate at 180 s. The dorsal aorta (DA), posterior cardinal vein (PCV) and site of laser injury (LI, arrows) are indicated.

We evaluated *Tg(itga2b:EGFP)* thrombocyte responses to venous laser injury in 5 dpf *fga^+/+^*, *fga*^*+/*Δ*19–56*^ and *fga*^Δ*19–56/*Δ*19–56*^ larvae. Initially, we verified the number of circulating thrombocytes was equivalent in 5 dpf larvae for each *fga* genotype. No significant differences were measured (Supplementary Figure S5). As for *fga*^−/−^ larvae (Figure 2), in *fga*^Δ*19–56/*Δ*19–56*^ thrombocytes largely failed to adhere or aggregate after PCV laser injury (Figure 6A,B). However, in contrast to *fga^+/−^* larvae which showed equivalent thrombocyte accumulation to *fga*^+/+^, *fga*^*+/*Δ*19–56*^ heterozygotes gave an intermediate thrombocyte binding and accumulation phenotype, with the fluorescence curve measured over time clearly intermediate between *fga^+/+^* and *fga*^Δ*19–56/*Δ*19–56*^ (Figure 6A). This demonstrates a dominant effect on thrombocyte activity for the mutated allele in *fga*^*+/*Δ*19–56*^. Examples for fluorescent thrombocyte activity in *fga^+/+^* and *fga*^Δ*19–56/*Δ*19–56*^ are given in Supplementary Videos 8 and 9, respectively. For video scaling, compare to Figure 6B.

### 2.5. A Zebrafish Model of Dysfibrinogenemia

**Figure 7:**
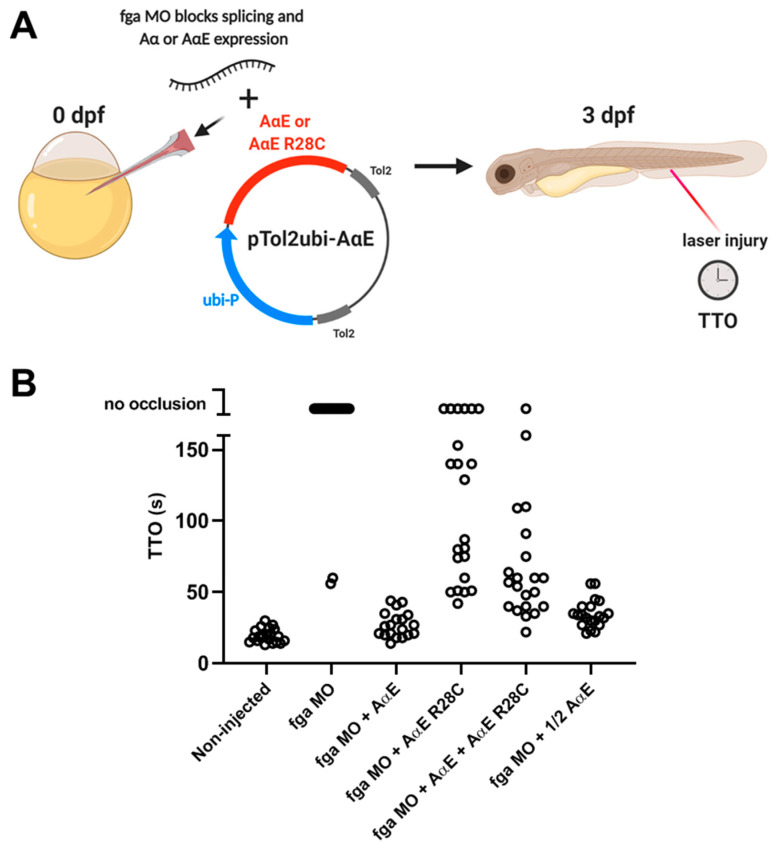
TTO measurements in fga morpholino knockdown embryos with transgenic expression of AαE, AαE R28C, or both, to model dysfibrinogenemia. (**A**) A scheme representing the experimental set-up. Early TU zebrafish embryos were injected with and without an fga mRNA-targeting morpholino (MO) in the absence or presence of Tol2 plasmids for expression of fibrinogen AαE, AαE R28C, AαE + AαE R28C or a half quantity of AαE (1/2 AαE). Tol2 transposase mRNA was also present within injection mixes (not shown). At 3 dpf the TTO assay was performed to measure venous thrombosis time. The MO inhibits endogenous fga mRNA splicing but cannot target the transgenically expressed AαE and AαE R28C mRNAs. (**B**) TTO data for each experimental group, each circle represents one larva (*n* = 19 to 22).

As our initial aim was to assess venous thrombosis in a model of dysfibrinogenemia, but targeted genome editing gave fga mRNA exon 2 skipping, instead of a missense mutation, we used an alternative approach (Figure 7A). TU background zebrafish embryos were microinjected with an antisense morpholino (MO) that targets splicing of the fga pre-mRNA and leads to >90% depletion in fga mRNA [39]. In the TTO assay with 3 dpf larvae, 18/20 MO-injected larvae gave no occlusion 3 min after laser injury, whereas all noninjected larvae gave occlusion (mean TTO 19.5 s, *n* = 21) (Figure 7B). In separate injection mixes, the MO was complemented with Tol2 transposon plasmid DNA and transposase mRNA for transgenic expression of the fibrinogen AαE chain cDNA or AαE R28C. The MO cannot target the cDNA sequence. The AαE chain is the predominant fibrinogen alpha chain isoform expressed in larval TU zebrafish [35] and was therefore chosen as the most relevant chain in the TTO assay.

Transgenic expression of AαE complemented MO knock-down, reversing the MO TTO phenotype with all larvae supporting venous occlusion and a mean TTO of 27 s (*n* = 19) (Figure 7B). Expression of AαE R28C permitted occlusion in 16/22 larvae but the TTO was prolonged compared to noninjected or AαE-injected larvae (88 s, *n*=16). To mimic the heterozygous state of patients with dysfibrinogenemia and the R35C mutation, we injected the MO with a 1:1 mix of AαE and AαE R28C plasmids. Venous occlusion was measured in 20/21 larvae, with shorter TTO than AαE R28C alone, but markedly prolonged TTO compared to AαE alone (mean TTO 62 s, *n* = 20). To control for the half quantity of AαE plasmid injected in the AαE + AαE R28C condition, we also injected ½ quantity of AαE plasmid with the MO and measured TTO at 3 dpf. All larvae supported occlusion with a mean TTO considerably shorter than the AαE + AαE R28C mix and marginally longer than the initial MO + AαE condition. This demonstrates the dominant effect of AαE R28C expression, seen when comparing the TTO in AαE + AαE R28C versus AαE alone, is not due to lower quantities of injected AαE plasmid in the mixed condition.

**Figure 8:**
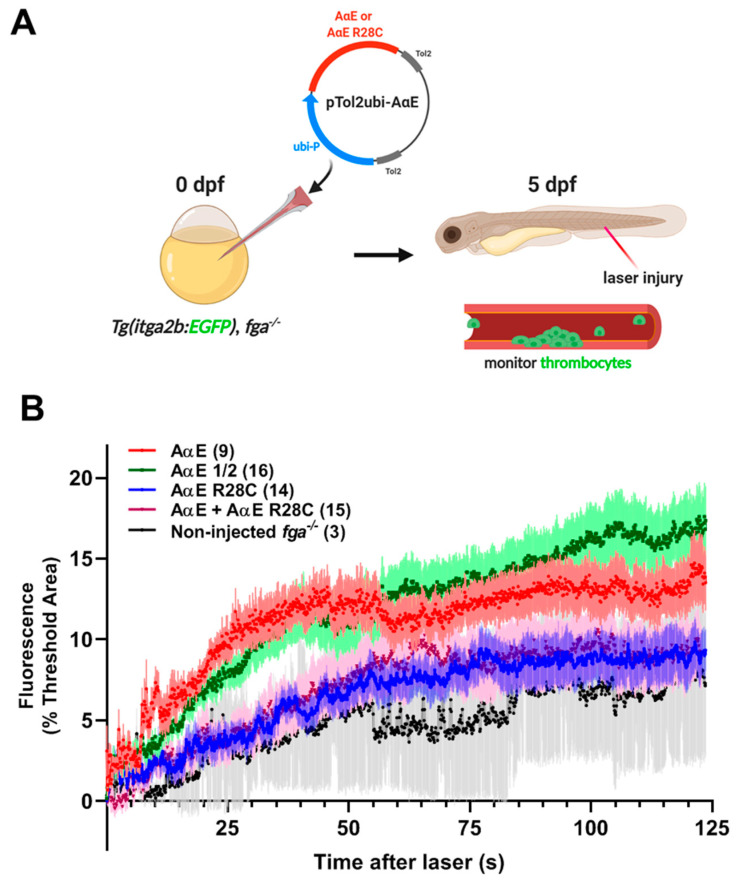
Fluorescent thrombocyte binding and aggregation after PCV laser injury in *fga^−/−^* larvae with transgenic AαE or AαE R28C expression: a model of dysfibrinogenemia. (**A**) A scheme representing the experimental setup. Early *Tg(itga2b:EGFP)*, *fga^−/−^* embryos were microinjected with Tol2 transgenesis plasmids for expression of fibrinogen AαE or AαE R28C, AαE + AαE R28C or a half quantity of AαE (AαE ½). Tol2 transposase mRNA was also present in injection mixes (not shown). (**B**) Fluorescent thrombocyte binding and adhesion at 5 dpf were monitored for each experimental group over time after PCV laser injury. Each line represents the mean fluorescence (+/−SEM). The number of larvae per group is shown in brackets.

To monitor the effect of the fibrinogen AαE R28C mutation on laser-induced thrombocyte adhesion and aggregation we used transgenic expression of AαE and AαE R28C in *fga^−/−^* larvae with the *itga2b:EGFP* transgene and fluorescent thrombocytes at 5 dpf (Figure 8A). The MO-based fga mRNA knock-down could not be used as it is transient and does not persist effectively to 5 dpf (RJF, unpublished). Expression of AαE increased markedly the initial adhesion and accumulation of thrombocytes to sites of venous laser injury compared to noninjected *fga^−/−^* larvae (Figure 8B), as reported previously [35]. Fluorescence curves for thrombocyte activity in larvae with transgenic expression of AαE R28C, or a mix of AαE + AαE R28C to mimic a heterozygous state, were largely similar to noninjected *fga^−/−^* controls, demonstrating a failure of AαE R28C to complement fibrinogen deficiency and a dominant effect of AαE R28C in the mixed condition. Injecting half the quantity of AαE to control for the amount of AαE in the AαE + AαE R28C condition gave a similar result to AαE, reiterating the dominant effect of AαE R28C on thrombocyte binding and accumulation at sites of venous laser injury.

## 3. Discussion

In this study we assessed the phenotype of larval zebrafish models of congenital fibrinogen disorders using laser-induced venous thrombosis and fluorescent thrombocyte adhesion and accumulation as the functional read-outs. We aimed to determine whether the experimental venous thrombosis phenotype of afibrinogenemia, a quantitative disorder, differs from that of dysfibrinogenemia—a disorder of fibrinogen quality.

The model we used for afibrinogenemia has been reported previously [25] and our TTO assay data are similar to those reported elsewhere [27,35]. The TTO in 3 dpf zebrafish larvae measures the propensity to thrombose after injury but is also a measure of clotting potential, as *fga^−/−^* animals fail to support clotting and subsequent thrombosis. In 5 dpf *fga^−/−^* larvae we observed a hint of the thromboembolic phenotype seen in afibrinogenemic patients [8]. Thrombocyte aggregates form, but can be mobile, partly explaining the flattened accumulation curves for fluorescent thrombocytes described. In the future it will be interesting to monitor the destination and fate of these aggregates, as a potential source of distant vessel occlusion.

Ferric chloride (FeCl_3_)-induced platelet thrombus formation persisted in the arterioles of fibrinogen-deficient mice [40], a result that may seem at odds with our data for 5 dpf *fga^−/−^* larvae where thrombocyte accumulation is markedly affected compared to control, and vessel occlusion not seen. Larval zebrafish thrombocytes potentially lack molecules present in adult mouse platelets that are necessary to support thrombosis in the absence of fibrinogen. There could also be differences in the vasculature or plasma which determine fibrinogen-dependency in laser-induced venous thrombosis in larval zebrafish compared to FeCl_3_-induced thrombosis in adult murine arterioles.

Despite the defect in laser-induced thrombocyte binding we describe, we have no evidence for spontaneous bleeding in *fga^−/−^* larvae. Perhaps residual thrombocyte activity at 5 dpf is enough to prevent bleeding, but we have not assessed this directly. The functional importance of thrombocytes in adult zebrafish hemostasis was recently highlighted by synthetic lethality detected in homozygous *fga* mutants with thrombocytopenia [27]. However, this was measured by survival rates over a year and is therefore not directly comparable to our laser-induced injury phenotype measured over a few minutes at a defined developmental age.

We initially encountered difficulties in modelling the R35C dysfibrinogenemia mutation. CRISPR-Cas9 based genome edits of *fga* led to a splicing defect in fga mRNA despite incorporation of the desired transmissible sequence changes. Engineering codon usage changes and an enzyme recognition sequence alongside the missense mutation (R28C) appeared to have disrupted the regulation of the usual fga mRNA splicing mechanism. While we were able to use the resulting mutant line as a model of hypodysfibrinogenemia (*fga*^*+/*Δ*19–56*^), we would not have seen the final outcome of genome editing had we not assayed the fga transcripts in our mutants. Caution should therefore be taken when basing mutant phenotype assignment based on an engineered genomic sequence change alone.

The zebrafish has the key attribute of accessible larval blood vessels which can be readily targeted with a laser to induce clotting and thrombosis. For the present study an obvious limitation is that the disorders we model are diagnosed in part by the concordance of plasma fibrinogen levels and activity. At present, to our knowledge, plasma fibrinogen cannot be measured accurately in larval zebrafish blood due to low blood volumes and a lack of methodology. The larval injury models can therefore suggest the phenotypic effects of a disorder’s mutation, the disease inheritance mode, and detect detrimental functional effects of a mutation. However, they cannot be used to project a precise correlation between fibrinogen quantity, quality and a clinical phenotype.

The mutated fibrinogen alpha chains produced in our proposed models of dysfibrinogenemia (R28C) and hypodysfibrinogenemia (Δ19–56) lack the Aα amino terminal sequence for effective thrombin-mediated cleavage and FpA release. While clots are still likely to occur via B:b knob-hole interactions [41], retention of FpA is known to affect fibrin polymerisation, change clot structure [37] and susceptibility to fibrinolysis [42]. The prolonged thrombosis phenotype we observed in both models is consistent with changes in fibrin polymerisation, and altered thrombocyte binding to injury sites may reflect changes in cellular interactions with an altered clot structure.

Thus far, the differences between the distinct model phenotypes we report are subtle. Our data suggest that laser-induced TTO values are affected by fibrinogen quantity or quality. This can be seen by the minor prolongation of mean TTO in *fga^+/−^* or *fga*^+/Δ19–56^, compared to *fga^+/+^* controls, and the longer TTO measured in fga-targeted morpholino knock-down larvae with AαE + AαE R28C expression, compared to AαE expression alone. The phenotype of binding and accumulation of fluorescent thrombocytes after laser injury is possibly more discriminatory between the models. Curves of fluorescence used to represent thrombocyte activity after laser injury were unaffected in *fga^+/−^* larvae compared to *fga^+/+^*, whereas thrombocyte accumulation was lowered in models of the heterozygous state in dysfibrinogenemia or hypodysfibrinogenemia.

This leads us to propose an expected zebrafish phenotype profile for models of quantitative versus qualitative disorders in our two laser injury assays. Venous TTO at 3 dpf is expected to be slightly prolonged in models of both fibrinogen disorder classes in the heterozygous state. Afibrinogenemia models fail to support venous occlusion, whereas models of qualitative disorder mutations in homozygosity (*fga*^Δ*19–56/*Δ*19–56*^ or expression of AαE R28C), that are detected in heterozygosity in patients, give some larvae where TTO is measurable and many others with no occlusion. In afibrinogenemia models, 5 dpf thrombocytes adhere and aggregate poorly to laser injury sites and have a propensity for embolism. This is a recessive trait; in heterozygous carriers of an allele which confers afibrinogenemia in homozygosity, thrombocyte activity is similar to wild-type controls. Models of qualitative congenital fibrinogen disorders are expected to demonstrate dominant negative effects on thrombocyte activity. Neither of the functional read-outs at their present resolution distinguish between the dysfibrinogenemia and hypodysfibrinogenemia models presented.

In the future we aim to use this preliminary guide to assess the phenotype of newly uncovered mutations linked to congenital fibrinogen disorders and take steps towards correlating the larval zebrafish model phenotypes with clinical indicators in patients.

## 4. Materials and Methods

### 4.1. Zebrafish

Adult zebrafish were maintained at 26 °C, pH 7.5, and 500 µS conductivity. Embryos from natural matings were raised at 28.5 °C. The *fga* mutant zebrafish, a model of afibrinogenemia, were described previously [25]. Genome edited zebrafish expressing fibrinogen Aα^Δ*19–56*^, were generated in the TU background. Zebrafish with the *itga2b:EGFP* transgene were a gift from Leonard Zon’s laboratory, Harvard Medical School. Experimentation was authorized by local veterinary authorities, authorization GE/161/19 (07.11.2019).

### 4.2. Production of Zebrafish Fibrinogen in Transfected Cells and Thrombin Cleavage

The human fibrinogen Aα R35C mutation prevents FpA cleavage by thrombin. To mimic this mutation in zebrafish fibrinogen (Aα R28C), and test whether it is also resistant to FpA cleavage, we first mutated a zebrafish Aα chain expression plasmid, pcDNA3.1-ZF-Aα, using the QuikChange II XL Site-Directed Mutagenesis Kit (Agilent, Santa Clara, CA, USA) and the oligonucleotides fgaR28C-F (5′GGACACAGTGGTGAACCCTTGCGGTGCTCGTCCTATTGAGC3′) and fgaR28C-R (5′GCTCAATAGGACGAGCACCGCAAGGGTTCACCACTGTGTCC3′). The mutation was confirmed by DNA sequencing. The wild-type or mutated Aα plasmids, were cotransfected into HEK-293T cells in 10 cm cell culture dishes with plasmids for the expression of zebrafish fibrinogen Bβ and γ chains, using Lipofectamine 2000 (Thermo Fisher Scientific, Walthum, MA, USA). A nontransfected control sample was also prepared. Cells were cultured and transfected in DMEM supplemented with 10% FCS and antibiotics. At 24 h posttransfection the culture medium was removed, cells washed with PBS and then cultured for a further 24 h in OptiMEM (Thermo Fisher Scientific, Walthum, MA, USA) without serum. Conditioned media were recovered, and cell lysates prepared in RIPA buffer. Samples of each were incubated with or without 0.5 U/mL human thrombin (Merck KGaA, Darmstadt, Germany) for 1 h at 37 °C and subjected to western blotting using rabbit anti-zebrafish Aα or Bβ antibodies, as described previously [25].

### 4.3. Generation of Zebrafish Producing Fibrinogen Aα^*Δ*19–56^

We aimed to produce a zebrafish line expressing fibrinogen Aα R28C. A CRISPR-Cas9 strategy was used. Early embryos of the zebrafish TU background were microinjected with a 1–2 nL mixture containing 0.5 ng/nL recombinant Cas9 nuclease (PNABio, Newbury Park, CA, USA), 250 pg/nL of a single guide RNA (sgRNA) with complementarity to zebrafish *fga* exon 2, 0.44 ng/nL of a single stranded oligonucleotide (ssODN, IDT, Leuven, Belgium) for template-mediated homology-directed repair (HDR), Danieau buffer (58 mM NaCl, 0.7 mM KCl, 0.4 mM MgSO_4_, 0.6 mM Ca(NO_3_)_2_, 5.0 mM HEPES pH 7.6) and phenol red as a tracer. The sgRNA was produced by in vitro transcription, using the MEGAshortscript kit (Thermo Fisher Scientific, Walthum, MA, USA), of a linearized plasmid based on pDR274 [43] that contains the template for *fga* exon 2 targeting introduced by cloning of annealed oligonucleotides (R28sg1F, 5′TAGGACACAGTGGTGAACCCTAG3′ and R28sg1R, 5′AAACCTAGGGTTCACCACTGTGT3′). pDR274 was a gift from Keith Joung (Addgene plasmid 42250; http://n2t.net/addgene:42250; RRID:Addgene_42250). The ssODN repair template was designed to mutate the zebrafish fibrinogen Aα chain Arginine 28 codon to Cysteine. It also introduced 3 codon usage changes to avoid retargeting of the edited sequence by sgRNA-guided Cas9. One of these also introduced an HpaI restriction site, useful for PCR-genotyping. The full ssODN sequence is given below with the HpaI site underlined, the Cysteine codon boxed and the codon usage changes in bold. The antisense sequence with respect to *fga* transcription was used:

5′CCATACCCAGTCATCATCGGTACACCCTGGCCATTCTTTTGTCTGGCAGGTGTCTTGTGCCTTGAAGCCGTGCTCAATAGGACGAGCGCCGCAAGGGTT**A**ACCAC**G**GT**A**TCCTCCTCGGCCTAAAAAGTAAGTACTTTATAAC3′

Microinjected F0 embryos were raised to adulthood and crossed with wild-type fish to identify a founder animal. F1 offspring were raised and genomic DNA from F1 embryos extracted and assayed for the R28C mutation by PCR genotyping and HpaI digestion, and DNA sequencing to confirm the mutation. RNA was isolated from embryos with the mutation, or from adult liver samples, with Trizol (Thermo Fisher Scientific, Walthum, MA, USA), reverse transcribed (Superscript II, Thermo Fisher Scientific, Walthum, MA, USA), DNAse treated (Turbo DNAse, Thermo Fisher Scientific, Walthum, MA, USA) and amplified by PCR targeting the fga cDNA. PCR products were cloned in pCRII TOPO (Thermo Fisher Scientific, Walthum, MA, USA) and sequenced. This demonstrated that fga exon 2 skipping occurred in transcripts where the Aα R28C codon was introduced, and encoded Aα^Δ*19–56*^.

### 4.4. Tol2 Plasmid Constructions

A plasmid for expression of the zebrafish fibrinogen AαE chain, under the control of a ubiquitin (*ubb*) promoter (subsequently labelled ubi), was prepared by Gateway cloning (Thermo Fisher Scientific, Walthum, MA, USA). Middle entry clones for AαE cDNAs were made using attB1- and attB2-tagged oligonucleotides to PCR amplify cDNAs and pDONOR221 (Thermo Fisher Scientific, Walthum, MA, USA) as a vector for recombination with PCR products, yielding pME-AαE. These were used in 4-way Gateway recombination reactions with pENTR5’_ubi, (Addgene plasmid # 27320; http://n2t.net/addgene:27320; RRID:Addgene_27320) [44], pDestTol2CG2-U6:gRNA (Addgene plasmid # 63156; http://n2t.net/addgene:63156; RRID:Addgene_63156) [45], both gifts from Leonard Zon, and p3E polyA from the Tol2kit [46]. The final plasmid for transgenesis was named pTol2ubi-AαE. A pTol2ubi-AαE R28C clone was prepared by site-directed mutagenesis, as described above for pcDNA 3.1 Aα R28C.

### 4.5. Microinjections for fga mRNA Knockdowns and Fibrinogen Alpha Chain Overexpression

Early zebrafish embryos were microinjected with approximately 1 nL of injection mixes. These contained Danieau buffer (58 mM NaCl, 0.7 mM KCl, 0.4 mM MgSO_4_, 0.6 mM Ca(NO_3_)_2_, 5.0 mM HEPES pH7.6), phenol red, and where described 2 ng of an *fga* exon1-intron1 splice site-targeting antisense morpholino (5′GCATTATATCACTCACCAATGCAGA3′, Genetools Inc., Philomath, OR, USA). For transgenic expression of AαE or AαE R28C, injection mixes included 25 ng of pTol2ubi-AαE or pTol2ubi-AαE R28C and approximately 35 ng of 5′capped, in vitro-polyadenylated Tol2 transposase mRNA. Where noted, injection included 12.5 ng pTol2ubi-AαE or a mixture of 12.5 ng pTol2ubi-AαE + 12.5 ng pTol2ubi-AαE R28C.

### 4.6. Genotyping

Mutations were detected by PCR-genotyping of adult tail fin clips or embryo lysates using *fga* oligonucleotides for PCR (fgaset1F, 5′AATGGCCTATGTTGGCAGAC3′ and fgaset1R, 5′CAGTGGTTATCAGCTGACAG3′) and restriction digests with BceAI for the previously-described *fga* mutants [25] or HpaI to detect *fga^Δ19–56^*.

### 4.7. Plasma Collection and Western Blots

Dilute plasma was prepared from wild-type adult fish and those with 1 or 2 copies of the mutated *fga* allele leading to expression of Aα^Δ*19–56*^ (*fga*^+/+^, *fga*^*+/*Δ*19–56*^, *fga*^Δ*19–56/*Δ*19–56*^), as described previously [25], a protocol adapted from [47]. Reduced sample preparations of dilute plasma were subjected to Western blotting with anti-zebrafish fibrinogen Aα (Covalab, Bron, France) and anti-zebrafish ceruloplasmin antibodies (Eurogentec, Liège, Belgium).

### 4.8. Laser-Mediated Thrombosis and Thrombocyte Accumulation in Zebrafish Larvae

Venous thrombosis in zebrafish larvae was assessed with two assays. The time-to-occlusion (TTO) was measured in seconds after laser injury laser of the posterior cardinal vein (PCV) in 3-day postfertilization (3 dpf) larvae [48]. In the second test, older, 5 dpf *Tg(itga2b:EGFP)* larvae with circulating fluorescent thrombocytes were subjected to a similar PCV injury and the thrombocyte fluorescence accumulation around the injury site recorded and measured. For both assays, larvae were anesthetized in Tricaine (170 μg/mL), placed on 0.22% low gelling point agarose on glass microscope slides and visualized with a Leica LMD microscope (Leica Microsystems, Wetzlar, Germany). The microscope used an HCX PL FLUOTAR L 20x/0.40 corr objective and a cryslas laser (max. pulse energy: 50 µJ, pulse frequency: 80 Hz, wavelength: 355 nm). Bright field images were acquired with a Leica LMD CC7000 camera and fluorescence with a Leica DFC 360 FX, using LMD and LAS-AF software, at room temperature. Thrombocyte activity image series were analyzed with MetaMorph Offline software, v.7.10.1.161 (Molecular Devices, LLC. San Jose, CA, USA). Thrombocyte numbers in 5 dpf larvae with the *fga*^+/+^, *fga*^*+/*Δ*19–56*^, *fga*^Δ*19–56/*Δ*19–56*^ genotypes were compared by counting *itga2b:EGFP*-positive cells flowing through the PCV over a 30 s period.

### 4.9. Statistical Analysis and Graphics

Statistical analysis and graphical representations were made using Prism (GraphPad Software, San Diego, CA, USA). Figure 7A and Figure 8A and part of Figure 1A were made with BioRender (https://app.biorender.com/).

## Data Availability

Data are available on request from the corresponding author.

## Supplementary Materials

Supplementary Materials can be found at https://www.mdpi.com/1422-0067/22/2/655/s1.

## Abbreviations

DA	Dorsal aorta
dpf	Days postfertilization
LI	Laser injury
MO	Morpholino
PCV	Posterior cardinal vein
TTO	Time to occlusion
