# Supplementary material for: Venous Thrombosis and Thrombocyte Activity in Zebrafish Models of Quantitative and Qualitative Fibrinogen Disorders
Source: Int J Mol Sci. 2021 Jan 11;22(2):655. doi: 10.3390/ijms22020655 (PMC7826895; doi:10.3390/ijms22020655)
Supplement: Supplementary file 1 [file ijms-22-00655-s001.zip › ijms-1049622 supplementary figures.pdf]

Supplementary data Fish et al.

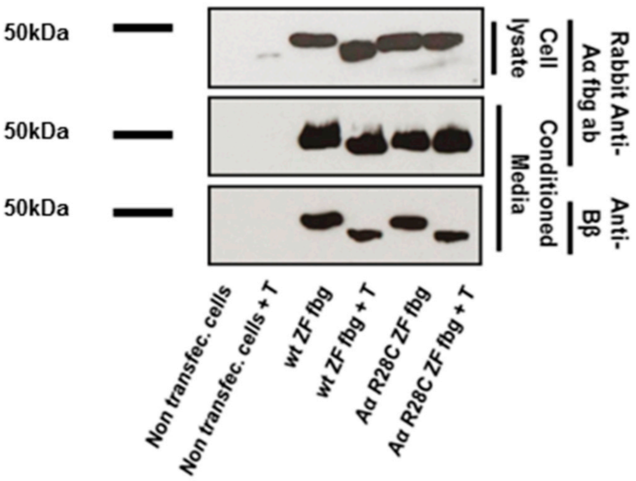

Supplementary Figure S1 –Thrombin cleavage of zebrafish fibrinogen and fibrinogen A*α* R28C. Immunoblot of zebrafish (ZF) fibrinogen (fbg) in cell lysates and conditioned media from transfected HEK-293T cells with and without thrombin (T) treatment. Anti-A*α* and anti-Bβ fibrinogen antibodies were used. Controls were non-transfected HEK-293T cells.

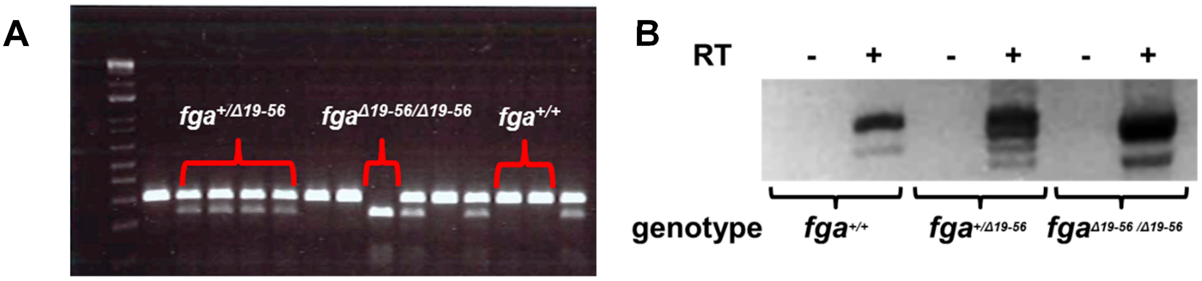

Supplementary Figure S2 – ***fga***$^{\Delta 19\text{-}56}$ **genotyping and RT-PCR from liver samples. (A)** PCR-genotyping individual fish using the restriction enzyme HpaI. After HpaI digestion, *fga*$^{+/+}$: 301 nucleotide (nt) band; *fga*$^{+/\Delta 19\text{-}56}$: 301 nt + 230 nt + 71 nt bands and *fga*$^{\Delta 19\text{-}56/\Delta 19\text{-}56}$: 230 nt + 71 nt bands. **(B)** RT-PCR of RNA from *fga*$^{+/+}$, *fga*$^{+/\Delta 19\text{-}56}$, and *fga*$^{\Delta 19\text{-}56/\Delta 19\text{-}56}$ liver samples with and without reverse transcriptase (RT + or –).

A

fga exon 2 – Predicted ESEs:

```
          ACACAG
       AGGACA                       CGTCCT
      GAGGAC                    CGCTCG
     GGAGGA                    GCGCTC
    AGGAGG                    GGCGCT
   GAGGAG                   GAGGCG
GCCGAG           GGTGAA    AGAGGC               CACGGC
GCCGAGGAGGACACAGTGGTGAACCCTAGAGGCGCTCGTCCTATTGAGCACGGCTTCAAGGCACAAGACACCTGCCAGACAAAAGAATGGCCAGGGTGTACCGATGATGACTGG
.........|.........|.........|.........|.........|.........|.........|.........|.........|.........|.........|....
         10        20        30        40        50        60        70        80        90        100       110
```

B

Edited _fga_ exon 2 – Predicted ESEs:

```
                                    CGTCCT
                                CGCTCG
      GAGGAT                   GCGCTC
     GGAGGA                   GGCGCT
    AGGAGG                   CGGCGC
   GAGGAG                   GCGGCG
GCCGAG                     TGCGGC               CACGGC
GCCGAGGAGGATACCGTGGTTAACCCTTGCGGCGCTCGTCCTATTGAGCACGGCTTCAAGGCACAAGACACCTGCCAGACAAAAGAATGGCCAGGGTGTACCGATGATGACTGG
.........|.........|.........|.........|.........|.........|.........|.........|.........|.........|.........|....
         10        20        30        40        50        60        70        80        90        100       110
```

Supplementary Figure S3 – Exon Splicing Enhancer Sequence prediction (ESES) in *fga* exon 2 from wild-type and after genome editing. A splicing element annotation tool (RESCUE-ESE Web Server - genes.mit.edu) was used to predict the Exonic Splicing Enhancer (ESE) sequences in *fga* exon 2 and in *fga* exon 2 with targeted genome editing as described. In *fga* exon 2, 15 ESE sequences were predicted. In the edited *fga* exon 2, 13 sequences were predicted. 5 ESE sequences that were present in the *fga* exon 2 are absent in the edited *fga* exon 2, and 3 new ESE sequences have appeared in the edited sequence. In yellow are nucleotide changes for codon usage, in blue is the nucleotide change to generate an HpaI cleavage site for genotyping, and in green is the codon change designed for the R28C mutation.

A

B

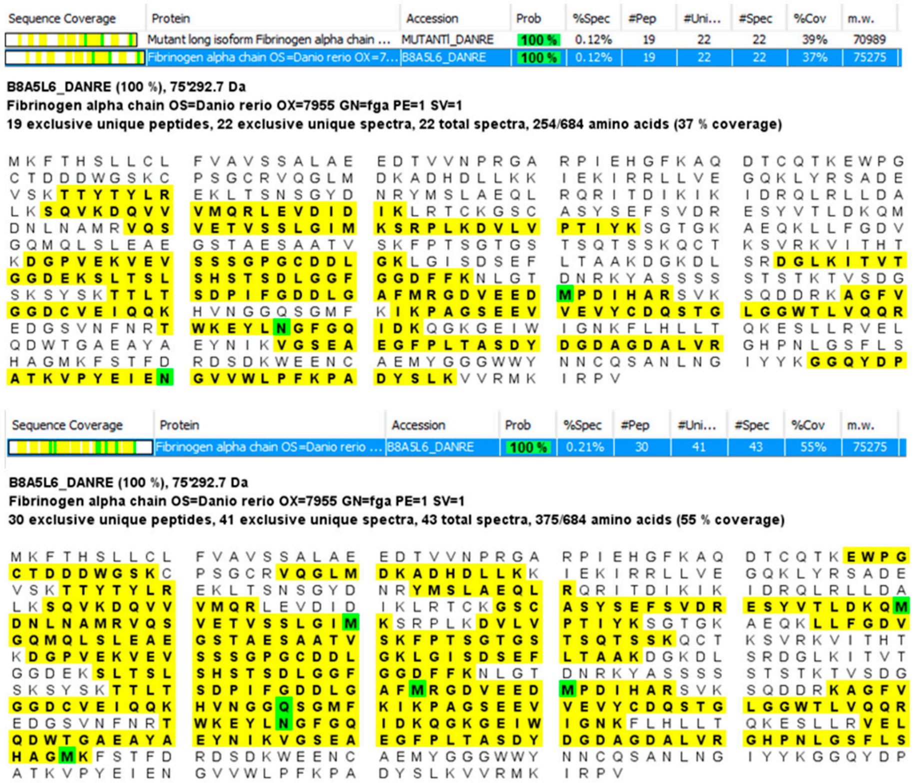

Supplementary Figure S4 – Protein identification by ESI-LC-MSMS. Protein identification by ESI-LC-MSMS on two zebrafish plasma samples to distinguish fibrinogen alpha chain from wild-type control and fibrinogen alpha chain from a genome edited mutant sample. After sample concentration, proteins were digested and peptides were analysed by nanoLC-MSMS using an easynLC1000 (Thermo Fisher) coupled with a Qexactive Plus mass spectrometer (Thermo Fisher). Database searches were performed with Mascot (Matrix Science) using Zebrafish Reference Proteome database (uniprot.org) supplemented with fibrinogen mutant sequences. Data were analysed and validated with Scaffold (Proteome Software) with 1% of protein FDR and at least 2 unique peptides per protein with a 0.1% of peptide FDR. **(A)** For the mutant: 323 proteins were identified. Fibrinogen alpha was identified with 19 exclusive unique peptides covering 37% of the protein sequence (yellow). **(B)** For the control sample: 388 proteins were identified. Fibrinogen alpha was detected with 30 exclusive unique peptides covering 55% of the protein sequence (yellow). However, in this sample, a specific peptide unique to the wild-type sequence of the fibrinogen Aα chain encoded by *fga* exon 2 was detected (EWPGCTDDDWGSK).

Supplementary Figure S5 – Circulating thrombocytes in 5dpf zebrafish larvae. The number of

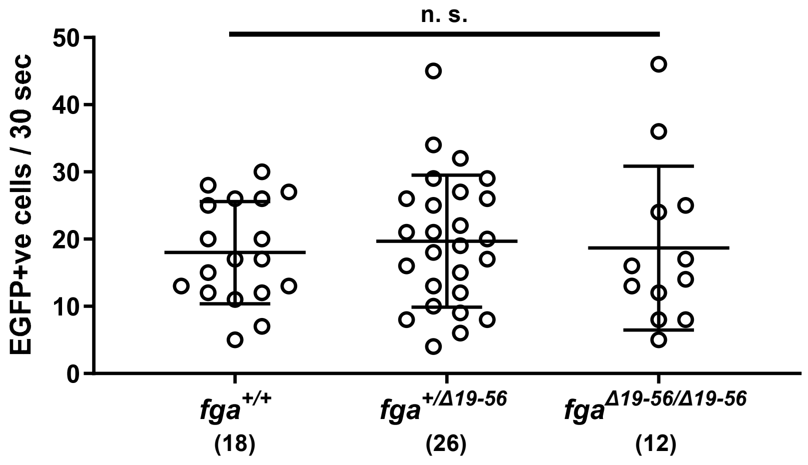

Tg(itga2b:EGFP) thrombocytes passing through the posterior cardinal vein in 30 sec (EGFP+ve cells / 30 sec) was monitored for *fga*$^{+/+}$, *fga*$^{+/\Delta 19\text{-}56}$ and *fga*$^{\Delta 19\text{-}56/\Delta 19\text{-}56}$ larvae at 5dpf. Unpaired t-test: *fga*$^{+/\Delta 19\text{-}56}$ vs. *fga*$^{+/+}$ p=0.5422; *fga* $^{\Delta 19\text{-}56/\Delta 19\text{-}56}$ vs. *fga*$^{+/+}$ p=0.8546; *fga*$^{+/\Delta 19\text{-}56}$ vs $^{\Delta 19\text{-}56}$ p=0.7832 (n. s. = not significant). The number of assessed larvae for each group is shown in brackets.
